# Peeling of secondary epiretinal membrane in uveitis–functional improvement to be expected?

**DOI:** 10.1186/s40942-025-00773-3

**Published:** 2025-12-09

**Authors:** Julia Schirrwagen, Verena Schöneberger, Claudia Brockmann, Thomas A. Fuchsluger, Friederike Schaub

**Affiliations:** 1 Department of Ophthalmology, Rostock University Medical Center, Doberaner Str. 140, 18057 Rostock, Germany; 2 Department of Ophthalmology, Medical Faculty and University Hospital Düsseldorf, Heinrich Heine University Düsseldorf, Düsseldorf, Germany

**Keywords:** Epiretinal membrane peeling, Uveitis, ILM peeling, Pars plana vitrectomy, Surgery, Cystoid macular edema

## Abstract

**Background:**

There is a paucity of information regarding the results of patients with uveitis and secondary epiretinal membrane (sERM) who undergo pars plana vitrectomy and membrane peeling. This study aims to analyse the functional and anatomical outcomes and possible prognostic factors of a large cohort of eyes with uveitis-associated sERM who underwent vitrectomy with epiretinal membrane peeling.

**Methods:**

The results of 76 eyes of 76 consecutive patients with uveitis-associated sERM who underwent pars plana vitrectomy with membrane peeling were analysed. The mean follow-up duration was 42.7 ± 47.9 months. Best-corrected visual acuity (BCVA) and central retinal thickness (CRT) before and after intervention were measured. Furthermore, demographic data, type of uveitis according to the Standardization of Uveitis Nomenclature (SUN) classification, benefit of additional peeling of the Membrana limitans interna (ILM), activity status of the uveitis at the time of surgery, lens status and postoperative complications were evaluated. Statistical tests included paired t tests, Wilcoxon signed-rank tests, Mann‒Whitney tests, and Kruskal‒Wallis H tests. Statistical significance was defined as p < 0.05; Holm‒Bonferroni correction was employed to address the cumulative risk of false-positive outcomes (type I error).

**Results:**

CRT improved from 421.2 ± 133.2 µm prior to surgery to 331.7±142.5 µm at the final follow-up (p = 0.069), whereas BCVA deteriorated from a mean of 0.49 ± 0.30 logMAR to 0.56 ± 0.60 logMAR in the overall cohort (p > 0.99). The rate of concomitant cystoid macular edema decreased from 42.4% to 34.3%.

**Conclusions:**

The indications for membrane peeling in patients with a secondary epiretinal membrane and uveitis should be considered carefully. Anatomical features can be positively influenced by pars plana vitrectomy with ERM peeling, whereas BCVA may only result in beneficial changes in carefully selected patients.

## Background

The epiretinal membrane (ERM) is a pathological membrane at the vitreoretinal interface [1] occurring as idiopathic ERM (iERM) and also a common complication in patients with uveitis, with an incidence of up to 54% [2–5]. Patients with longer-lasting uveitis have a greater risk of developing a secondary ERM (sERM) [2]. ERM incidence varies across uveitis subtypes (28.1% in anterior uveitis, 57.0% in intermediate uveitis, and 43.4% in posterior uveitis and panuveitis) [2]. This distribution likely reflects ERM formation at the vitreoretinal interface [2, 6, 7]. The membrane consists of multiple cell types on the membrana limitans interna (ILM), including contractile cells [1], although the pathophysiology is still not fully understood. Contraction can lead to a variety of symptoms, such as decreased visual acuity, metamorphopsia, blurred vision, diplopia and even loss of central vision [2, 8, 9], although some ERMs can be asymptomatic and are diagnosed accidentally [10]. Diagnosis relies on a combination of clinical examination and optical coherence tomography (OCT). ERMs present as irregular and hyperreflective layers above the ILM and can be associated with wrinkling of the retina [11].

In patients with uveitis and sERM, several factors contribute to reduced best-corrected visual acuity (BCVA). Complications such as cataract formation, retinal detachment [12], or cystoid macular edema (CME) [4], which itself is common in uveitis [13], can impair vision. Additional determinants include the duration and stage of the ERM, as well as the duration and etiology of uveitis [14].

Symptomatic patients are typically treated with pars plana vitrectomy and ERM peeling, with optional additional ILM peeling. While surgical outcomes are well established for iERM [15], data on uveitis-associated sERM remain limited and inconsistent [16]. Previous studies have been limited by small sample sizes, heterogeneous patient populations, and variable follow-up durations, leaving uncertainty about long-term functional and anatomical outcomes as well as prognostic factors such as uveitis type, disease activity, and OCT features [4, 15–18]. Moreover, the role of ILM peeling in this setting remains debated: while it may reduce recurrence, several studies have reported no additional functional benefit [15, 17, 19, 20].

This study aims to address these gaps by analysing the long-term outcomes of vitrectomy with membrane peeling in a large cohort of patients with uveitis-associated sERM, with particular attention given to functional and anatomical outcomes and potential prognostic factors.

## Methods

**Fig. 1:**
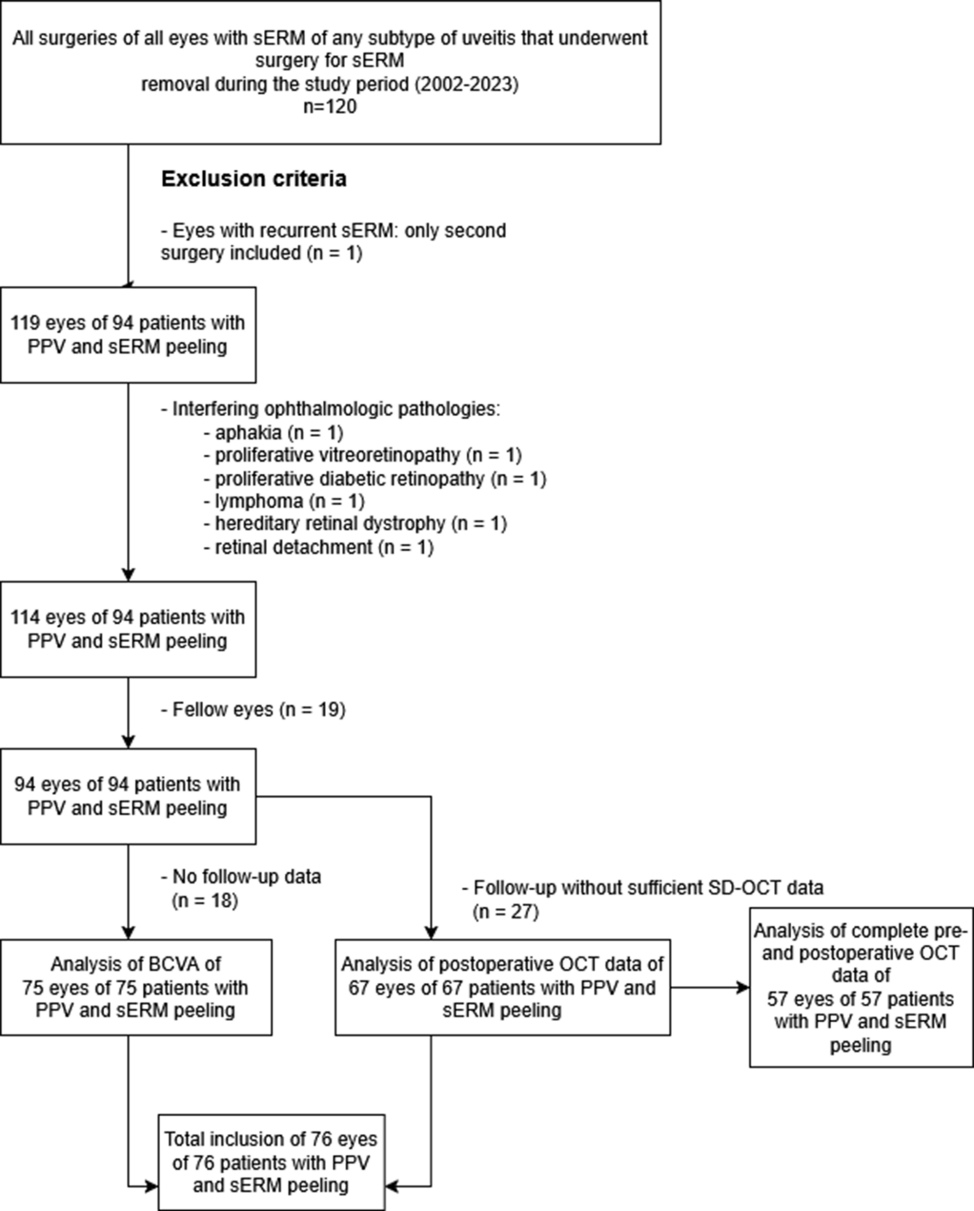
Flow chart of patient selection illustrating the number of patients screened, excluded, and included in the main analysis. The diagram summarizes how many eyes were eligible for the BCVA analysis and how many had OCT data pre- and postoperative for structural evaluation. The final cohort size used for the main outcome analysis is also displayed

This retrospective, single-center study included all consecutive patients who underwent pars plana vitrectomy with epiretinal membrane peeling due to secondary epiretinal membrane resulting from uveitis (*n* = 120 surgeries in 119 eyes of 100 patients) at the Department of Ophthalmology, University Medical Center Rostock, Germany. The data were collected over the course of 21 years, from November 2002 to April 2023. Data from OCT examinations were available from 2009 onwards. The primary outcomes of this study were changes in best-corrected visual acuity (BCVA) and central retinal thickness (CRT); secondary analyses focused on prognostic factors and postoperative complications. Uveitis was classified according to the SUN criteria into anterior, intermediate, posterior, or panuveitis [21]. The diagnosis was based on slit-lamp and fundus examinations and, when indicated, was supplemented by multimodal imaging, including OCT. The indications for ERM surgery in uveitis patients were based on individual clinical decisions in each case. The main criteria included relevant visual impairment (reduced BCVA), the presence of disturbing symptoms such as metamorphopsia, and/or progressive anatomical changes on OCT (e.g., increasing central retinal thickness or tractive macular edema). All consecutive eyes with sERM of any subtype of uveitis that underwent surgery for sERM removal during the study period were considered (see Fig. 1). The exclusion criteria included a lack of follow-up (*n* = 18) and the presence of additional severe ophthalmologic pathologies such as aphakia (*n* = 1), proliferative vitreoretinopathy (*n* = 1), proliferative diabetic retinopathy (*n* = 1), lymphoma (*n* = 1), hereditary retinal dystrophy (*n* = 1) and retinal detachment (*n* = 1). If both eyes of a patient met the established criteria, one eye was randomly selected for subsequent investigation (*n* = 19). In one patient with recurrent ERMs, only the second surgery was considered. Patients were included if BCVA, postoperative OCT or both data were available. BCVA data were available for 75 eyes, and OCT data were available for 67 eyes, resulting in a total of 76 eyes included.

The collected data included demographics, uveitis etiology, and activity status, which were defined as ongoing local or systemic steroids or immunosuppressants at surgery. Furthermore, data on uveitis therapy, lens status, surgical details, postoperative complications, functional and anatomical outcomes, and additional ophthalmological diseases were recorded. Visual acuity was measured with decimal charts and converted to the logarithm of the minimum angle of resolution (logMAR). Follow-up intervals were defined as short-term (2–6 months), midterm (6–12 months), and long-term (final follow-up) intervals to capture early, intermediate, and late outcomes, corresponding to the standard postoperative schedule at our clinic.

The study was conducted in accordance with the International Conference on Harmonization for Good Clinical Practice (ICH-GCP) and at all times adhered to the Declaration of Helsinki (2000). Favourable opinions were obtained from the local Institutional Review Board (IRB No. A 2022–0124).

### OCT examinations

Morphological parameters were examined via macular spectral-domain OCT (Spectralis®, Heidelberg Engineering GmbH, Heidelberg, Germany). The specific scan protocol was a custom raster scan pattern with 19 or 37 sections (512 A-scans each) in a 30° × 20° field of view. OCT examinations were performed before and at each postoperative visit during follow-up. At each time point, central retinal thickness (CRT) and macular volume (MV) were measured. Therefore, the Early Treatment Diabetic Retinopathy Study (ETDRS) grid (6 mm × 6 mm square grid) was first manually replotted to the foveal center, and errors in device-provided segmentation lines were corrected on all the scans enclosed within the central subfield. Visible deviations in the segmentation lines were corrected manually. CRT (μm) measurements were derived from the topographic map of the macular cube scan for a 1 mm foveal area extracted from the ETDRS grid. The MV (mm^3^) was also extracted from the topographic map of the macular cube scan. Additionally, the thickness of the ERM was measured, and qualitative changes, including the presence of hyperreflective foci and focal or broad adhesions of the epiretinal membrane, were recorded.

### Surgical technique

Surgery was performed under retrobulbar anaesthesia in the majority of the patients. General anaesthesia was indicated for patients who were unable to cooperate due to dementia or severe anxiety. The surgical approach was 23-gauge or 25-gauge 3-port pars plana vitrectomy performed by experienced vitreoretinal surgeons. In the case of coexisting cataracts, PPV was combined with cataract extraction (standard clear cornea bimanual phacoemulsification followed by monofocal posterior chamber, in-the-bag lens implantation). In the majority of cases, after removal of the vitreous and conventional ERM peeling, complete removal of the ILM via forceps was performed. The choice to peel the ILM was made at the surgeons’ discretion, without a standardized protocol.

ILM peeling was performed at the posterior pole up to the major vascular arcade and near the optic disc. For staining of the ERM and ILM, Brilliant Peel® (Geuder Company GmbH, Heidelberg, Germany) or Twin Blue (AL.CHI.MI.A. S.R.L., Italy) was used at the discretion of the surgeons. In early years, vitrectomies were also performed without dye. ERM and, if performed, ILM removal was followed by flooding the vitreous chamber with either room air or a 25% sulfur hexafluoride (SF6) air mixture and removing the trocars. Only in patients with tractive retinal detachment was silicone oil chosen for endotamponade. Subconjunctival or parabulbar dexamethasone or triamcinolone was applied in selected cases at the surgeons’ discretion. Systemic steroid therapy or immunosuppressive therapy was also administered or continued depending on the activity status of the uveitis. Postoperative regimens included prednisolone acetate administered at least five times daily and tapered over four weeks and a fluoroquinolone administered four times daily for two weeks. In the case of active uveitis, additional topical or systemic steroids were administered or, more frequently as needed.

### Statistics

Statistical analysis was performed via IBM SPSS Statistics software (version 29.0.2.0 for Windows, SPSS, Inc., Chicago, IL). Continuous variables were assessed with the Shapiro‒Wilk test. Normally distributed data are presented as the means and standard deviations; nonnormally distributed data are described as medians and interquartile ranges (IQRs). We performed subgroup analyses after excluding statistical outliers, which were identified according to the standard boxplot definition (values > 1.5×IQR from the quartiles). The statistical tests employed for two-way paired data were the paired t test and the Wilcoxon signed-rank test. For univariate comparisons, the Mann‒Whitney test was used to compare two independent variables. To compare continuous variables across more than two independent groups, the Kruskal‒Wallis H test was applied. All the statistical comparisons were performed relative to the respective baseline value of each group and subgroup. BCVA (logMAR) showed non-normal distribution (Shapiro–Wilk). Accordingly, non-parametric tests were used. Due to the large sample size, mean BCVA values are reported for interpretability, with median and IQR provided in Table 1. Statistical significance was defined as *p* < 0.05. Given the number of statistical comparisons, we used the Holm‒Bonferroni correction [22] to control the cumulative risk of false-positive results (type I error). All *p* values are presented after correction.

**Table 1:** Demographic data of the whole study cohort and descriptive statistics of functional and morphological parameters

Demographic data of the whole cohort	Before sERM peeling	After sERM peeling (last follow-up)	p value
Sex (female) n = 76	60.6%		
Age at timepoint of surgery n = 76	63.8 ± 13.3 (range 22 – 88 years)		
BCVA (logMAR)	0.49 ± 0.30 0.4 (0.5)* n = 76	0.56 ± 0.61 0.3 (0.4)* n = 75	> 0.99
Central retinal thickness (µm)	421.2 ± 133.2 n = 59	331.7 ± 142.5 n = 67	0.046
Macular volume (mm^3^) according to EDTRS	9.9 ± 2.0 n = 59	8.5 ± 1.8 n = 67	0.046
Uveitis classification (according to SUN) (%) n = 76	Uveitis anterior: 15.8% Uveitis intermedia: 47.4% Uveitis posterior: 30.3% Panuveitis: 6.6%		
Endotamponade n = 76	Balanced Salt Solution: 10.4% Air: 55.8% Gas (SF6 25%): 32.5% Silicone oil: 1.3%		

## Results

### Baseline characteristics

This study examined 76 eyes of 76 patients who underwent vitrectomy and membrane peeling due to secondary epiretinal membrane resulting from uveitis. The majority of patients (60.6%) were female, and 51.0% had right eyes. The mean age at surgery was 63.8 ± 13.3 years (ranging from 22 to 88 years), and the mean follow-up duration was 42.7 ± 47.9 months (median 29.5 months, ranging from 1 to 189 months). A total of 53.9% (*n* = 41) of the eyes were phakic at baseline.

The distribution of uveitis types according to the SUN classification [21] was as follows: 15.8% (*n* = 12) of patients had anterior uveitis, 47.4% (*n* = 36) had intermediate uveitis, 30.3% (*n* = 23) had posterior uveitis, and 6.6% (*n* = 5) had panuveitis (see Table 1).

The infectious etiology identified in 9.2% (*n* = 7) of the cases included toxoplasmosis (2.6%, *n* = 2), yersiniosis (2.6%, *n* = 2), borreliosis (1.3%, *n* = 1), lues (1.3%, *n* = 1) and chlamydia (1.3%, *n* = 1). A total of 5.3% (*n* = 4) of patients were HLA-B27 positive, whereas 21.1% (*n* = 16) were negative. For 75.0% (*n* = 57) of patients, no data regarding HLA-B27 were available.

### Surgical details

In the majority of cases (93.4%, *n* = 71), both the ERM and the ILM were peeled. Five different surgeons performed surgery during the 21-year period. The decision to peel the ILM was left to the surgeon’s discretion; no standardized protocol was applied.

In 35.5% (*n* = 27) of the surgeries, the epiretinal membrane was coloured with a dye to make it more visible before peeling.

A total of 53.9% (*n* = 41) of patients received subconjunctival dexamethasone at the end of the surgery, and 34.2% (*n* = 26) received a combination of subconjunctival dexamethasone and intravenous prednisolone. A total of 6.6% (*n* = 5) of the patients received subconjunctival triamcinolone, and 2.6% (*n* = 2) received a combination of subconjunctival triamcinolone and 250 mg of intravenous prednisolone. For 2 patients, no data were available.

At the time of the surgical procedure, uveitis was active in 13.2% (*n* = 10) of the patients.

### Functional outcome

**Fig. 2:**
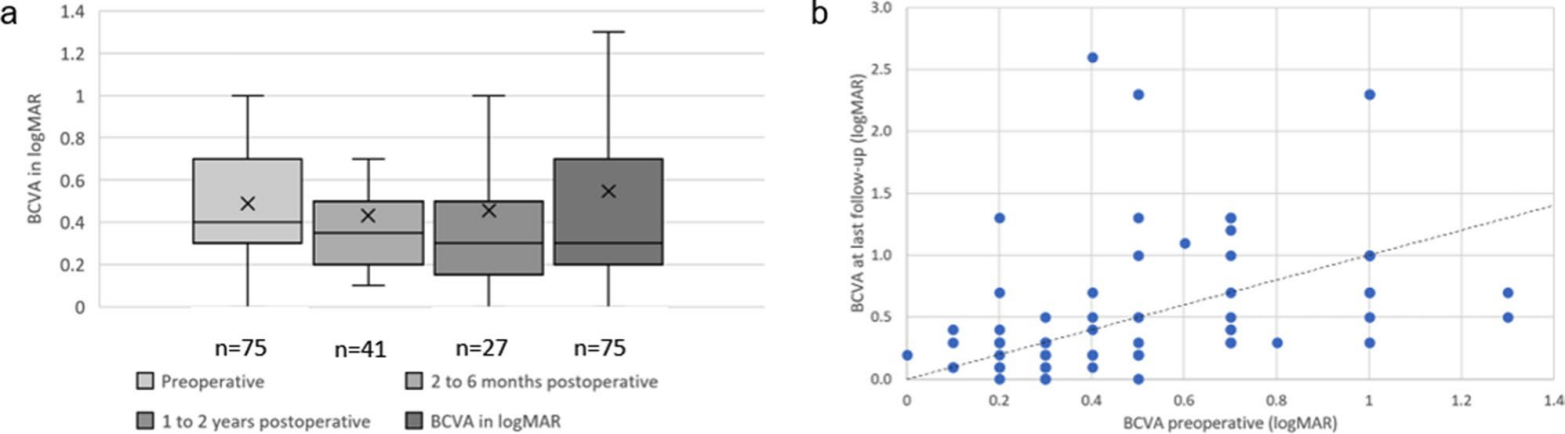
Functional results. 1a: development of visual acuity. 1b: scatter plot of best-corrected visual acuity before and after surgery. 2a: development of visual acuity. Boxes represent the first quartile, median (solid lines), mean (cross) and third quartile values. 2b: scatter plot of best-corrected visual acuity before and after surgery. The x-axis represents visual acuity prior to surgery, and the y-axis represents visual acuity after surgery at the last follow-up (42.7 ± 47.9 months, range from 1 to 189 months) after ERM peeling in uveitis patients. BCVA: best corrected visual acuity. logMAR: logarithm of the minimum angle of resolution. Below the diagonal dashed black line represents improvement in BCVA postoperatively

Data on visual outcome were available for 75 eyes. A total of 37 eyes (49.3%) improved in visual outcome, 13 eyes (17.3%) remained unchanged, and 25 eyes (33.3%) worsened over a mean follow-up of 42.7 ± 47.9 months. BCVA showed a non-normal distribution; detailed distribution metrics (median/IQR) are provided in table 1. The BCVA before surgery was 0.49 ± 0.30 logMAR in the overall cohort. During the follow-up period between two and six months after surgery, BCVA improved to 0.43 ± 0.30 logMAR (*p* > 0.99). In the period between six months and one year after surgery, it decreased to 0.53 ± 0.50 logMAR (*p* > 0.99). At the final follow-up (42.7 ± 47.9 months), it decreased to 0.56 ± 0.60 logMAR (*p* > 0.99) (see Figure 2a). All changes in visual function were statistically insignificant for the overall cohort.

Patients with BCVA improvement at the last follow-up had poorer initial BCVA (0.54 ± 0.31 logMAR) than patients with deteriorated BCVA (0.44 ± 0.27 logMAR, *p* > 0.99) (see Figure 2b).

There was no difference in functional outcome at the last follow-up between female (BCVA 0.59 ± 0.63 logMAR) and male patients (0.53±0.57 logMAR; *p* > 0.99). BCVA at baseline and logMAR gain at the last follow-up were slightly negatively correlated (Spearman’s ρ = −0.23, *p* > 0.99), indicating that patients with poor initial BCVA tended to have greater logMAR gains.

*N* = 3 of the patients with uveitis anteriorly improved visual acuity at the last follow-up examination (59.0 ± 45.4 months), whereas 47.2% (*n* = 17) of the patients with uveitis intermedia (follow-up 36.1 ± 51.1 months), 47.8% (*n* = 11) with uveitis posterior (follow-up 38.9 ± 37.5 months) and 80.0% (*n* = 4) (follow-up 67.8 ± 68.7 months) with panuveitis improved (see Table 2).

**Table 2:** Development of the BVCA in 75 patients with different types of uveitis, with peeling of the ERM and peeling of both the ERM and the ILM and in phakic and pseudophakic eyes

Best corrected visual acuity	n = 75	Preoperative (logMAR)	Follow-up 2–6 months after surgery (logMAR)	Follow-up 6–12 months after surgery (logMAR)	Last Follow-up (logMAR)
Uveitis anterior	11	0.44 ± 0.25 n = 11	0.43 ± 0.3 p > 0.99 n = 3	0.65 ± 0.84 p > 0.99 n = 6	0.87 ± 0.4 p > 0.99
Uveitis intermedia	34 (2 outliers)	0.48 ± 0.27	0.38 ± 0.24 p > 0.99 n = 19	0.42 ± 0.25 p > 0.99 n = 8	0.44 ± 0.34 p > 0.99
Uveitis posterior	18 (5 outliers)	0.42 ± 0.24	0.27±0.11 p > 0.99 n = 12	0.41±0.49 p > 0.99 n = 7	0.23±0.14 p > 0.99
Panuveitis	5	0.54 ± 0.30	0.93 ± 0.40 p > 0.99 n = 4	0.70 ± 0.50 p > 0.99 n = 4	0.46 ± 0.54 p > 0.99
ERM peeling	4 (1 outlier)	0.80 ± 0.24	0.67 ± 0.55 p > 0.99 n = 3	0.70 ± 0.71 p > 0.99 n = 2	0.48 ± 0.26 p = 0.43
ERM+ILM peeling	59 (11 outliers)	0.44 ± 0.29	0.36 ± 0.23 p > 0.99 n = 34	0.41 ± 0.34 p > 0.99 n = 21	0.35 ± 0.30 p = 0.98
Phakic eyes	12 (1 outlier)	0.64 ± 0.33	0.32 ± 0.13 p > 0.99 n = 5	0.37 ± 0.24 p > 0.99 n = 3	0.32 ± 0.15 p = 0.18
Phakic eyes, cataract surgery during follow-up	27 (1 outlier)	0.40 ± 0.26			0.38±0.40 p > 0.99
Pseudophakic eyes	31 (4 outliers)	0.48 ± 0.29	0.34 ± 0.19 p > 0.99 n = 17	0.58 ± 0.42 p > 0.99 n = 10	0.50 ± 0.38 p > 0.99

A total of 53.9% (*n* = 41) of the eyes were initially phakic; of these, 68.3% (*n* = 28) underwent cataract surgery following the procedure after a mean time of 22.8 ± 31.0 months. BCVA improved in 76.9% (*n* = 10) of phakic eyes, in contrast to 57.1% (*n* = 16) of the initially phakic eyes that underwent cataract surgery during the follow-up period. Improvement was observed in 25.7% (*n* = 9) of the pseudophakic eyes (Table 2). In pseudophakic eyes, visual acuity appeared worse at the final follow-up (0.51 ± 0.30 to 0.71 ± 0.68 logMAR; *p* = 0.14). However, the variability within this subgroup was considerable (SD = 0.68). After exclusion of statistical outliers (defined as values > 1.5×IQR), this difference was no longer present, indicating that the apparent decline was driven by a small number of extreme values rather than a systematic effect. To ensure better comparability, outliers were consistently excluded across the following subgroup analyses.

The BCVA results for patients who underwent combined peeling of the ERM and ILM, as well as peeling of the ERM alone, yielded similar outcomes. Owing to the limited number of patients with ERM peeling alone, we refrained from conducting a statistical comparison of these groups (see Table 2).

BVCA improved in 60.0% of all eyes with active uveitis at the time of surgery (*n* = 10), with a mean improvement from 0.69 ± 0.31 logMAR to 0.58 ± 0.42 logMAR (*p* > 0.99). In contrast, eyes with inactive uveitis (*n* = 65) improved by 41.5%, with an overall increase in BCVA from 0.46 ± 0.29 logMAR to 0.58 ± 0.63 logMAR (*p* > 0.99). The occurrence of active uveitis at the time of surgery had no significant effect on the final visual outcome in the present study (*p* > 0.99). In 70% of patients with active uveitis, uveitis was found to be well controlled postoperatively. This was defined as the absence of increased therapy or clinical signs of inflammation, such as cells in the anterior or posterior chamber. In contrast, among patients without active uveitis at the time of surgery, 84.6% demonstrated well-controlled uveitis postoperatively.

The procedure was performed by five different surgeons. No statistically significant difference was observed in the BCVA at the last follow-up among patients who underwent surgery performed by different surgeons (*p* > 0.99).

### Anatomical outcome

**Fig. 3:**
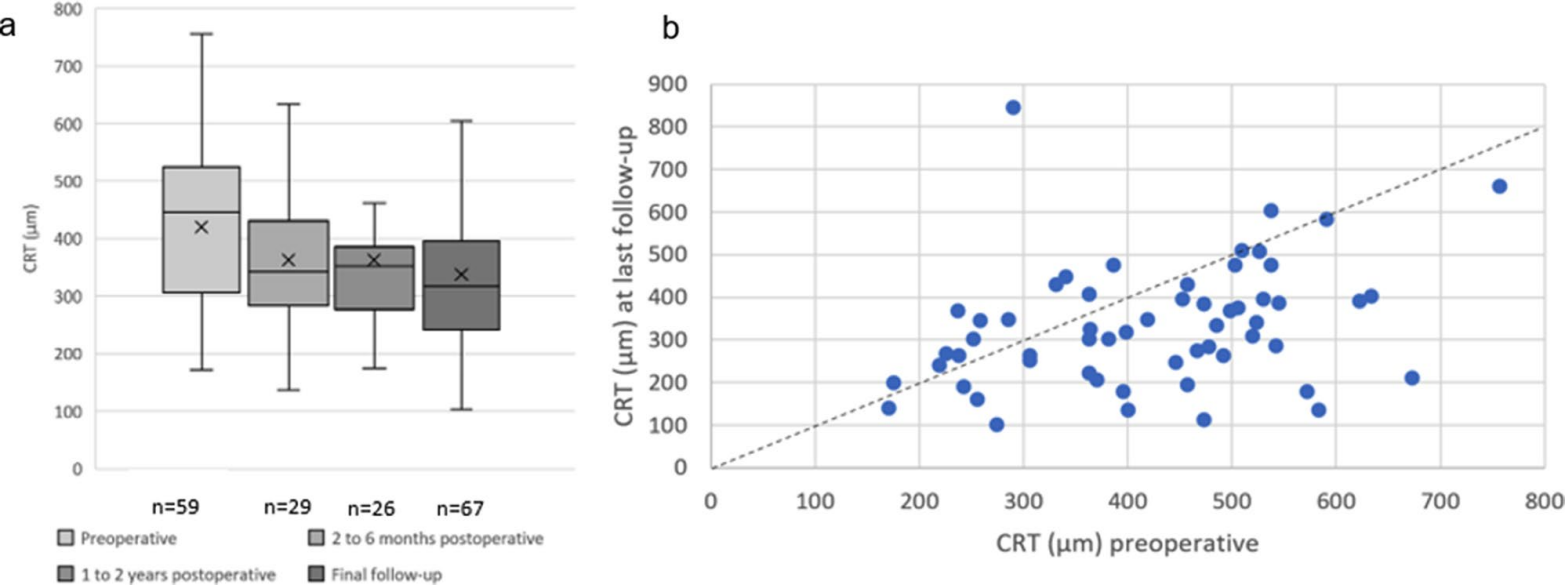
Anatomical outcome. **a**: development of CRT. **b**: scatter plot of pre- and postoperative CRT. **a**: development of CRT. CRT was measured preoperatively, between two and six months post-operatively, one to two years post-operatively, and at the final follow-up (42.7±47.9 months). Boxes represent the first quartile, median (solid lines), mean (cross) and third quartile values. **b**: scatter plot of pre- and postoperative CRT. The x-axis represents the CRT prior to surgery, the y-axis represents the CRT after surgery at the last follow-up (42.7±47.9 months, range from 1 to 189 months) after ERM peeling in uveitis patients. Below the diagonal dashed black line represents improvement in CRT postoperatively. CRT: central retinal thickness

Preoperative OCT data were available for 59 patients, whereas postoperative OCT data were available for 67 patients. Consequently, pre–post OCT comparisons were feasible only in a subset of patients. The mean thickness of the epiretinal membrane was 19.2 ± 3.3 µm. The central retinal thickness was 421.2 ± 133.2 µm at baseline. Two to six months after surgery, the CRT decreased to 362.7 ± 135.2 µm (*p* = 0.58); between six months and one year, it decreased further to 321.76 ± 98.70 µm (*p* > 0.99) at the last follow-up examination and remained stable at 331.7 ± 142.5 µm (*p* = 0.069) (see Fig. 3).

**Fig. 4:**
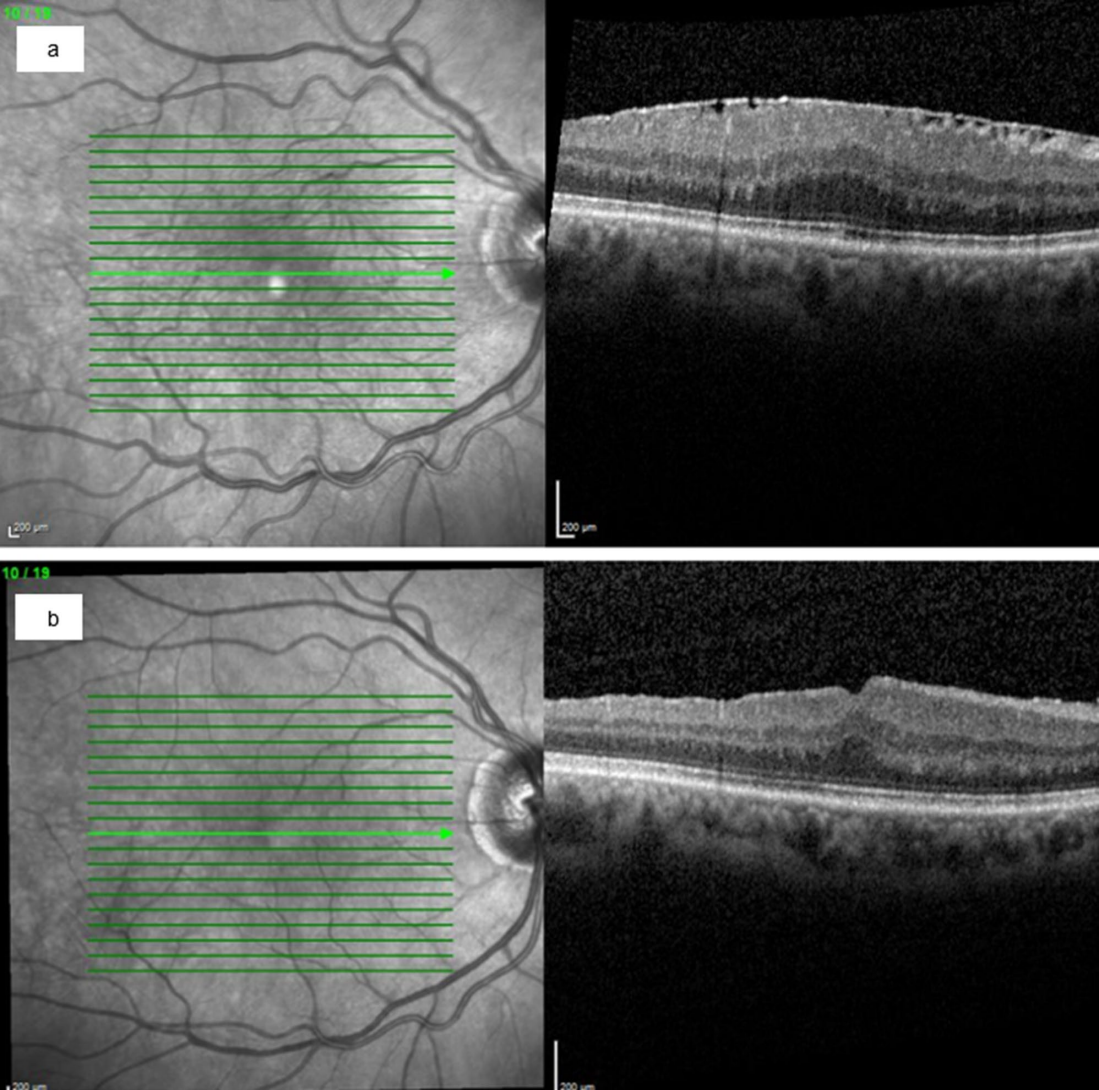
Exemplary case. Male, age 64 years at the time of surgery, uveitis anterior, right eye, with secondary epiretinal membrane (**a**) before and (**b**) three years after membrane peeling. The central retinal thickness was reduced from 530 µm to 395 µm, and the BCVA increased from 0,1 logMAR to −0,1 logMAR

Figure 4 shows the OCT findings and morphological changes of an exemplary case before (Fig. 4a) and three years after (Fig. 4b) peeling of the sERM and ILM.

42.4% (*n* = 25) had concomitant cystoid macular edema (CME) at baseline. We did not differentiate between the origins of CME, whether it was induced by ERMs or uveitis. For 67 patients, OCT data after surgery were available. During the follow-up period, 19.4% (*n* = 13) of those patients presented with new-onset CME, with the onset occurring at 6.7 ± 6.3 months following surgery. A total of 34.3% (*n* = 23) of all patients with an OCT examination had CME at the last examination (37.6±43.2 months post-operatively). In 16.4% (*n* = 11) of those patients, CME had been identified prior to surgery, whereas 11.9% (*n* = 8) developed new CME with no prior sign of this condition. Four patients (6.0%) with postoperative CME had no OCT prior to surgery. In 52.0% (*n* = 13) of patients with CME at baseline, it resolved postoperatively.

We did not observe a difference in visual outcome among patients according to different qualitative OCT parameters. There was no difference in BCVA between patients with and without disruption of the ellipsoid zone (*p* > 0.99) or between patients with and without posterior vitreous detachment (*p* > 0.99). The BCVA improved in 30.8% of patients with disruption of the ellipsoid zone, whereas 48.9% of patients without disruption improved. The BCVA improved in 30.9% of patients with posterior vitreous detachment, whereas improvement in BCVA in 43.9% of patients without posterior vitreous detachment was observed. BCVA at the final follow-up and the thickness of the epiretinal membrane were not correlated (Spearman’s ρ = −0.07, *p* > 0.99).

Eyes with CME at baseline had greater CRT than did eyes without CME (*p* > 0.99). Preoperatively, a moderate correlation between BCVA and CRT was observed (Spearman’s ρ = 0.38, *p* > 0.99) (see Table 3). Postoperatively, CRT decreased in eyes with preoperative CME (*p* = 0.069), whereas in eyes without CME, CRT was not significantly lower (*p* > 0.99). At the final follow-up, CRT did not differ significantly between eyes with and without baseline CME (*p* > 0.99), although the mean CRT reduction was greater in eyes with baseline CME (−189.8 ± 136.5 µm vs. −21.5 ± 142.4 µm, *p* = 0.069). BCVA decreased independently of CME status, with no significant difference between the groups at the last follow-up (*p* > 0.99). We categorized eyes with complete OCT data (*n* = 57) into four CME subgroups: persistent CME, resolved CME, new-onset CME, and without CME. The subanalysis showed distinct anatomical and functional patterns across these groups (see Table 4). Eyes with resolved CME demonstrated the most favorable course, with clear anatomical improvement and significant visual gain. Persistent CME was associated with reduced retinal thickening but no functional recovery, while new-onset CME showed neither anatomical nor visual improvement. Eyes without CME exhibited anatomical improvement but stable visual acuity. A weak postoperative correlation between BCVA and CRT persisted in the whole cohort (Spearman’s ρ = 0.25, *p* > 0.99).

**Table 3:** Spearman-correlations between BCVA and CRT in the overall cohort and subgroups

Comparison	Subgroup	Spearman’s ρ (Baseline)	p-value	Spearman’s ρ (Last follow-up)	p-value
Whole cohort					
BCVA vs. CRT	-	0.38	> 0.99	0.25	> 0.99
ΔBCVA vs. ΔCRT	-	ρ = 0.039		p > 0.99	
BCVA pre-post correlation	-	ρ = 0.56		p < 0.069	
CRT pre-post correlation	-	ρ = 0.35		p = 0.49	
CME					
BCVA/CRT	Eyes with initial CME	0.081	> 0.99	−0.137	> 0.99
BCVA/CRT	Eyes without initial CME	0.34	> 0.99	0.443	0.54
Uveitis activity					
BCVA/CRT	Eyes with active uveitis	−0.29	> 0.99	0.86	0.44
BCVA/CRT	Eyes without active uveitis	0.43	0.13	−0.2	> 0.99

**Table 4:** Analysis of pre- and postoperative BCVA and CRT for patients with CME

Subgroup	CRT (µm) Before sERM peeling	CRT (µm) After sERM peeling (last follow-up)	p-value	BCVA (logMAR) Before sERM peeling	BCVA (logMAR) After sERM peeling (last follow-up)	p-value
Persistend (*n* = 18)	516.7	343.8	> 0.99	0.50	0.71	> 0.99
Resolved (*n* = 5)	539.2	288.6	> 0.99	0.5	0.25	0.44
New Onset (*n* = 13)	384.2	390.5	> 0.99	0.45	0.55	> 0.99
Never (*n* = 21)	334.14	295.3	0.54	0.36	0.38	> 0.99

Eyes without baseline CME showed a moderate correlation between BCVA and CRT, in contrast to eyes with preoperative CME. Uveitis activity influenced patterns: in eyes with active inflammation, postoperative anatomical improvements were more likely accompanied by functional gain. Overall, change–change analysis confirmed that CRT reduction did not reliably predict BCVA improvement (see Table 3).

No statistically significant difference was observed when the mean CRT at the last follow-up of patients operated on by different surgeons was compared (*p* > 0.99).

## Complications

The incidence of complications after surgery was low (see Table 5) and included the recurrence of uveitis in 12 patients (15.7%). The recurrence of uveitis was characterized by anterior or posterior chamber cells and intensification of anti-inflammatory therapy. Further complications included recurrence of the epiretinal membrane in 6.6% (*n* = 5) of patients. A total of 5.6% (*n* = 4) of patients with combined peeling of the ILM and ERM had this complication, whereas 20.0% (*n* = 1) of patients with only peeling of the ERM experienced recurrence. Other complications included new-onset cystoid macular edema, rhegmatogenous retinal detachment and postoperative endophthalmitis. Initially, 41 patients were phakic. A total of 68.3% (*n* = 28) of those patients underwent cataract surgery after vitrectomy. A mean period of 22.4 ± 30.4 months passed between membrane peeling and cataract surgery.

**Table 5:** Complications after surgery

Complication	n	% (of the whole cohort n = 76)
Cataract surgery	28	36.8%
New-onset cystoid macular edema	13	17.1%
Recurrence of uveitis	12	15.7%
Recurrence of epiretinal membrane	5	6.6%
Rhegmatogenous retinal detachment	3	3.9%
Endophthalmitis	1	1.3%

## Discussion

This study evaluated the long-term functional and anatomical outcomes of pars plana vitrectomy with membrane peeling in 76 patients with uveitis-associated secondary epiretinal membranes (sERMs), with an additional focus on potential prognostic factors, including age, sex, and uveitis type.

In contrast to the findings of most previous reports [4, 15, 16, 18, 23], no overall improvement in the mean BCVA was observed in our cohort. This is likely explained by the higher baseline visual acuity and the inclusion of patients with heterogeneous preoperative conditions, including pseudophakia, cataract progression, and older age. Nevertheless, 49.3% of patients experienced BCVA improvement, with the greatest gains observed in eyes with poorer baseline BCVA. This finding is consistent with previous studies indicating that lower preoperative visual acuity is associated with greater postoperative gain [4, 10, 11, 14, 16]. Patients with poor baseline BCVA may have sustained advanced retinal damage but are also more likely to achieve functional gain from surgery owing to greater potential for improvement. Surgical benefits may additionally arise from the resolution of retinal traction and CME following membrane peeling [4, 6]. In contrast to idiopathic ERMs, baseline BCVA had a less pronounced prognostic impact on uveitis-associated sERM [17], likely due to ceiling effects, coexisting inflammatory changes, and cohort heterogeneity. Eyes with very good baseline BCVAs were generally not considered for surgery in our study because of the increased risk of recurrence of ERMs. Several studies have reported functional and anatomical improvements after ERM removal. Coassin et al. reported improved BCVA (from 0.73 to 0.42 logMAR) in 26 eyes with well-controlled uveitis [16]. Cristescu et al. demonstrated improvement in BCVA and CRT in 29 eyes, independent of uveitis activity or ILM peeling. CME occurrence was reduced [15]. Yap et al. reported BCVA improvement from 20/60 to 20/40 in 44 of 216 uveitic eyes that underwent ERM peeling, with vision loss mainly due to cataract progression and persistent CME [4]. Rao et al. noted a trend toward improved BCVA and CRT after combined ERM and ILM peeling in 17 eyes [18]. In contrast, Tanawade et al. did not observe overall BCVA improvement, emphasizing that surgery is most beneficial in eyes with macular traction or CME, whereas advanced macular pathology limits recovery [17].

In our cohort, BCVA values showed a non-normal distribution. Non-parametric tests were used for inference; however, the large sample size allowed mean BCVA values to serve as robust summary measures. Reporting means also supports comparability with existing literature and improves clinical interpretability.

In our cohort, no sex-related differences in outcomes were observed; only younger age at surgery correlated weakly with greater logMAR gain. BCVA outcomes did not differ significantly across uveitis types, with only nonsignificant trends toward improvement in panuveitis (*p* > 0.99). Our findings suggest that uveitis type does not significantly influence visual outcome after surgery, which is consistent with the findings of Coassin et al [16]. Overall, correlations between BCVA and CRT were weak, indicating that macular thickness alone does not reliably reflect visual function in uveitis-associated ERM. Subgroup analyses showed that a moderate structure–function relationship persisted only in eyes without baseline CME, while this association was lost in eyes with CME, underscoring the functional impact of edema. Uveitis activity also influenced correlations, with a strong postoperative relationship emerging exclusively in eyes with active inflammation, suggesting that structural recovery may translate into functional gain predominantly when inflammatory activity is present. The absence of a meaningful correlation between ΔCRT and ΔBCVA further confirms that anatomical improvement does not necessarily translate into visual recovery, highlighting the multifactorial nature of postoperative outcomes in this population.

We included patients with both ERM and ILM peeling as well as patients with ERM peeling alone. Owing to the retrospective design, surgical reports from five different surgeons were reviewed to identify ILM peeling (*n* = 71). In 38.0% (*n* = 27) of the patients, a dye was used to visualize the ERM; in the remaining patients, ILM peeling could not be definitively confirmed. BCVA outcomes were similar between combined ERM/ILM and ERM-only peeling, but the small number of ERM-only cases precluded statistical comparison. The decision to peel the ILM was surgeon dependent, as no standardized protocol was in place. While the role of ILM peeling remains debated [19, 24], several studies report no additional benefit for functional outcomes [15, 17, 19, 20]. In line with these findings, given the lack of significant differences between surgeons, the absence of a uniform ILM peeling protocol is unlikely to bias the results. However, ILM removal may reduce ERM recurrence [19, 24]. Consistent with this finding, recurrence occurred in 5.6% of the eyes with combined ERM/ILM peeling, whereas it occurred in 20.0% of the eyes with ERM peeling alone. In pseudophakic eyes, visual acuity seemed to decline at the final follow-up; however, this finding was accompanied by considerable variability. After exclusion of statistical outliers, the apparent difference was no longer present, indicating that the initial observation was likely driven by a few extreme values rather than a systematic effect. For consistency and better comparability, outliers were excluded across all subgroup analyses regarding BCVA. An improvement in BCVA was observed in phakic patients, whereas pseudophakic eyes and those that underwent cataract surgery showed no substantial change. Phakic eyes had poorer baseline BCVA, suggesting greater potential for improvement. Cataract progression and subsequent surgery likely contributed to a reduced BCVA, along with postoperative complications such as CME, posterior capsular opacification, pseudopakic retinal detachment, and late intraocular lens dislocation. Moreover, pseudophakic eyes are known to exhibit elevated levels of inflammatory mediators in the vitreous, even without uveitis. While the impact of these mediators on postoperative complications remains unclear, inflammatory reactions are the main cause of CME and can further impair vision. In our study, active uveitis was defined as the need for ongoing corticosteroid therapy at the time of surgery. Eyes with active uveitis at surgery showed greater BCVA improvement than inactive patients did, largely due to lower baseline BCVA. The final outcomes, however, did not differ significantly (*p* > 0.99). This finding suggests that disease activity per se does not limit surgical benefit. Intraoperative removal of inflamed tissue and inflammatory mediators may contribute to favourable results [25]. Postoperatively, uveitis was well controlled in 70% of active patients and 84.6% of inactive patients, with the difference likely reflecting more extensive systemic therapy in the active group. Surgery in eyes with active uveitis is generally avoided, as most authors recommend performing PPV during inactive uveitis (free of active inflammation for at least 3 months prior to surgery) to reduce the risk of postoperative inflammatory events, CME, and suboptimal visual recovery; typically, perioperative corticosteroids or immunomodulatory therapy are used to minimize these risks [16–18, 25–28]. Nevertheless, PPV may be justified in selected urgent cases with active inflammation, such as severe visual impairment, progressive macular pathology, or insufficient response to medical therapy, and may even help to reduce intraocular inflammatory mediators, though the risk of complications remains higher [15, 17, 18, 25, 26, 28]. In our cohort, surgery in eyes with active uveitis was performed only under these urgent indications. Given the heterogeneity in definitions of “active” versus “inactive” uveitis and the lack of standardized protocols, further prospective studies using SUN-based activity grading are needed to clarify optimal timing of PPV in uveitis-associated ERM. Anatomical outcomes were consistently favourable. CRT decreased in all patients (*p* = 0.069), which is consistent with prior studies [15, 16]. CME, present in 42.4% of patients at baseline, resolved in 52.0% of patients after surgery, persisted in 16.4%, and developed de novo in 11.9% of patients. These findings are in line with reports that membrane peeling reduces CME through traction release and removal of inflammatory mediators [10, 14, 29]. ERM contraction increases CRT [15], which can decrease after membrane peeling, partly because of the resolution of CME. CME is a common complication of both uveitis and ERM [29]. and can also occur postoperatively (Irvine–Gass syndrome) [30], potentially causing severe vision loss [31]. Vitrectomy with the ERM peel may improve CME by removing inflammatory mediators [32]. and reducing traction. Previous studies reported CME reduction after surgery [15, 17]. In our cohort, 42.4% of the eyes had CME at baseline, 34.3% at the last follow-up; 16.4% had residual CME, and 11.9% had newly developed CME. A total of 52.0% of the initial CME cases resolved postoperatively. The persistently high rate of CME likely contributes to the limited functional gain observed after ERM peeling. Eyes with preoperative CME had greater baseline CRT and experienced greater anatomical improvement postoperatively, yet this did not translate into superior BCVA outcomes. The weak correlation between postoperative BCVA and CRT indicates that CME is a relevant factor influencing visual recovery but is not the sole determinant. When examining CME subgroups, these dynamics became even more apparent. Eyes in which CME resolved, although the smallest subgroup, showed the most favorable postoperative course, with both anatomical improvement and meaningful visual gain. In contrast, persistent CME limited functional recovery despite reduced retinal thickening, and new-onset CME was associated with neither anatomical nor visual improvement. Eyes without CME demonstrated consistent CRT reduction but minimal BCVA change, likely reflecting a ceiling effect due to better baseline acuity. Together, these subgroup-specific patterns underscore that postoperative CME behavior is a key modulator of functional outcome, even though the global BCVA–CRT correlation remains weak. These findings are consistent with the notion that structural retinal changes, along with CME, collectively limit the potential for visual improvement in uveitis-associated sERM. The visual outcomes did not differ significantly on the basis of preoperative OCT features, such as ellipsoid zone disruption or posterior vitreous detachment. However, patients without these changes were more likely to improve, suggesting that extensive outer retinal damage may limit recovery and underscoring the need for further studies to identify prognostic OCT biomarkers. Postoperative complications were comparable to those in previous reports [15, 16, 18, 23]. Cataract surgery (36.8%) was the most common, followed by uveitis and/or ERM recurrence, retinal detachment, and endophthalmitis. A meta-analysis of idiopathic ERMs similarly identified cataract surgery as the predominant complication [33]. Notably, the complication rates reported in the previous study were lower than those reported in some prior studies on uveitic ERMs, possibly reflecting the importance of controlling inflammation. Our study reflects real-life outcomes in a heterogeneous patient population. While including a broad range increases variability, it provides a comprehensive view of functional and anatomical results. Exploratory analyses were performed to identify potential prognostic indicators, even if large differences were not always expected. The study is limited by its retrospective design over 21 years and relatively small overall sample size, resulting in small subgroups, particularly for panuveitis and sole ERM peeling. Variability in surgical techniques, dyes, and instrumentation was present but allowed assessment of their potential impact. Although this limits broad generalizability, it mirrors real-world clinical diversity. Uveitis activity at the time of surgery was defined on the basis of ongoing corticosteroid therapy, as documentation of clinical findings was inconsistent across the study period. A control group without ERM peeling could not be included since surgery is the only established treatment and is typically performed only when vision or quality of life is significantly affected. Systematic evaluation of perioperative steroid prophylaxis was not feasible because of the heterogeneity of the cohort, and the interval between the last episode of active uveitis and surgery could not be reliably assessed from referrals or clinical records. OCT analysis focused on CRT and macular volume; pre-post comparisons were possible in only 59 patients. Structural outer retinal changes were associated with less BCVA improvement, highlighting the need for further studies to identify prognostic OCT biomarkers. Our CME subgroup assessment revealed relevant heterogeneity: eyes with resolved CME demonstrated the most favorable functional postoperative course, whereas persistent or new-onset CME limited postoperative visual gain, and eyes without CME showed primarily anatomical rather than functional benefits. This supports the importance of incorporating CME dynamics into surgical decision-making. However, we did not distinguish between inflammatory and tractional CME mechanisms, which coexist in real-world uveitis. This overlap introduces interpretative complexity and limits conclusions about the individual contributions of ERMs and uveitis. Additional limitations include surgery by multiple surgeons, an unknown duration of ERM and uveitis, and overall limited follow-up. Nonetheless, to the best of our knowledge, this represents the largest cohort of uveitis patients with sERM analysed to date.

## Conclusion

Favourable functional outcomes were linked to poor baseline BCVA, younger age and postoperative CME resolution. Uveitis type (SUN classification) and activity at surgery did not affect final vision. In contrast, eyes with good preoperative vision, persistent or new-onset CME, or outer retinal changes limiting potential for functional recovery derived little benefit from surgery. ILM peeling reduced ERM recurrence but did not improve visual outcomes. Favourable anatomical outcomes were observed throughout the cohort [34].

## Data Availability

The data sets generated during and/or analyzed during the current study are available from the corresponding author upon reasonable request.
